# Stabilization of third-order differential equation by delay distributed feedback control

**DOI:** 10.1186/s13660-018-1930-5

**Published:** 2018-12-12

**Authors:** Alexander Domoshnitsky, Shirel Shemesh, Alexander Sitkin, Ester Yakovi, Roman Yavich

**Affiliations:** Department of Mathematics, Ariel University, Ariel, Israel

**Keywords:** Exponential stability, Stabilization, Delay differential equations, Cauchy function, W-transform

## Abstract

There are almost no results in mathematical literature on the exponential stability of third-order delay differential equations. One of the main purposes of the paper is to fill this gap. We propose an approach to the study of stability for third-order delay differential equations.

On the basis of these results, new possibilities of stabilization by delay feedback input control are proposed.

## Introduction

Stability of the first- and second-order functional differential equations were intensively studied in last decade (see, for example, [1–4] and the bibliography cited therein). Essentially less recent publications study third-order equations. The book by Seshadev Padhi and Smita Pati [5] demonstrates a new wave of the interest in the theory of third-order differential equations. Note that the previous book by Greguš [6] devoted to third-order equations was published more than 30 years ago. Various physical models based on third-order equations were presented in the recent book [5], let us start with them. Differential equations of the form

1.1 $$ x^{\prime \prime \prime }+a(t)x^{\prime \prime }+b(t)x^{\prime }(t)+c(t)x(t)=f(t) $$

arise in the analysis of entry-flow phenomenon. A problem of hydrodynamics was studied in many branches of engineering [7]. In [8] an integro-differential equation of the third-order modeling the steady flow of water in a long rectangular tank, oscillating horizontally near a resonant frequency, was studied. Note also the results of [9] on this object. The model describing the ionic mechanisms underlying the initiation and propagation of action potentials in the squid giant axon was proposed by Nobel Prize laureates of 1963 Alan Llyod Hodgkin and Andrew Huxley. A reduced version of this model was proposed by Nagumo (see for example [10]), suggesting a third-order differential equation as a model which presents many of the futures of the Hodgkin–Huxley equations.

Various applications of the third-order equations are based on the use of the delay feedback control for stabilization and this leads to the analysis of the asymptotic properties and stability of delay differential equations. We choose the control in the form

$$ u(t)=-\sum_{i=0}^{2}\sum _{j=1}^{m}p_{ij}(t)x^{(i)}\bigl(t- \tau _{ij}(t)\bigr), $$

where $p_{ij}(t)$ and $\tau _{ij}(t)$ are essentially bounded measurable functions for $j=1,\ldots,m$, $i=0,1,2$, where $\tau _{ij}(t)\geq 0$. In almost all real systems, we have $\tau _{ij}(t)>0$ since delay appears in receiving signal and in reactions on this signal.

Adding this control $u(t)$ in the right-hand side of equation (1.1), we arrive at the equation

$$ x^{\prime \prime \prime }+a(t)x^{\prime \prime }+b(t)x^{\prime }(t)+c(t)x(t)+\sum _{i=0}^{2}\sum_{j=1}^{m}p_{ij}(t)x^{(i)} \bigl(t-\tau _{ij}(t)\bigr)=f(t). $$

Various results on stability of third-order delay differential equations were presented in [11–20]. All noted results on stability were based on the method of Lyapunov’s functions. Results on stability based on the analysis of the characteristic equations for *n*th-order delay differential equations, which are quasipolynomials in the case of delay equations, were obtained in the well known books [21, 22]. In this paper we propose an absolutely different approach to the study of the exponential stability of third-order delay differential equations. Our approach is based on the idea of Azbelev’s *W*-transform presented in the book [23] (see Chapter 5) and developed then in [13].

As an example, let us consider the simplest model of ship stabilization [24, 25]. The equation

1.2 $$ Ix^{\prime \prime }(t)+hx^{\prime }(t)=-K\psi (t), $$

where *I*, *K*, *h* are corresponding constants, $I>0$ and $K>0$, can describe the ship dynamics. Here $x(t)$ is the ship deviation angle and $\psi (t)$ is the turning angle of the rudder. Following [22] (see p. 4), we can make the following steps. Assume that the change of rudder angle $\psi (t)$ is governed by the automatic helmsman rule

1.3 $$ T\psi ^{\prime }(t)+\psi (t)=\alpha y(t)+\beta y^{\prime }(t),\quad T>0, $$

where $y(t)$ is a measured value of the ship deviation angle, and *α*, *β* are the helmsman parameters. In practice, we can assume that $y(t)=x(t-\tau )$. Using the representation of the general solution of Eq. (1.3)

1.4 $$ \psi (t)=\frac{1}{T} \int _{0}^{t}e^{-\frac{1}{T}(t-s)} \bigl\{ \alpha y(s)+ \beta y^{\prime }(s) \bigr\} \,ds+e^{-\frac{1}{T}t}\psi (0), $$

substituting this representation into Eq. (1.2) and then differentiating, one arrives at the stability analysis of the third-order delay equation

1.5 $$ TIx{^{\prime \prime \prime }}(t)+(Th-I)x^{\prime \prime }(t)-hx^{\prime }(t)+K\beta x^{\prime }(t-\tau )+K\alpha x(t-\tau )=0. $$

We have to choose the helmsman parameters *α* and *β* to guarantee the exponential stability of Eq. (1.5).

The paper consists of the following sections. In Sect. 2, we formulated known results which are used in the proofs. In Sect. 3, auxiliary results on the Cauchy function for ordinary differential equations of the third order are obtained. In Sect. 4, the main results about stability of third-order delay differential equations are formulated. In Sect. 5, we prove the main theorem about stability. Conclusion, discussion of results and open problems are presented in Sect. 6.

## Preliminaries

Let us consider the following homogeneous equation:

2.1 $$ x^{\prime \prime \prime } ( t ) +\sum_{i=0}^{2}\sum _{j=1}^{m}p_{ij}(t)x^{(i)} \bigl(t-\tau _{ij}(t)\bigr)=0,\quad t\in [t_{0},\infty ), $$

and the corresponding non-homogeneous equation

2.2 $$ x^{\prime \prime \prime } ( t ) +\sum_{i=0}^{2}\sum_{j=1}^{m}p_{ij}(t)x^{(i)}\bigl(t-\tau _{ij}(t)\bigr)=f(t),\quad t\in [t_{0},\infty ), $$

where

2.3 $$ x(\xi )=\varphi (\xi ), \qquad x^{\prime }(\xi )=\psi (\xi ),\qquad x^{\prime \prime}(\xi )=\eta (\xi ) \quad \mbox{for }\xi < t_{0}, $$

and $p_{ij},\tau _{ij,}f:[0,\infty )\rightarrow \mathbb{R},\varphi ,\psi ,\eta :(-\infty ,0)\rightarrow \mathbb{R} $ are essentially bounded measurable functions for $j=1,\ldots,m$, $i=0,1,2$, where $\tau _{ij}(t)$ are nonnegative measurable bounded functions, without lost generality we assume that $t_{0}\geq 0$.

### Definition 2.1

We say that Eq. (2.1) is exponentially stable if there exist positive numbers *γ* and *N* such that

2.4 $$ \max_{t\geq 0} \bigl\{ \bigl\vert x(t) \bigr\vert , \bigl\vert x^{\prime }(t) \bigr\vert , \bigl\vert x^{\prime \prime }(t) \bigr\vert \bigr\} \leq Ne^{-\gamma (t-t_{0})}\underset{\xi \in (-\infty ,0)}{\mbox{ess} \sup } \bigl\vert \varphi (\xi ),\psi (\xi ),\eta (\xi ) \bigr\vert , $$

where *γ* and *N* do not depend on $t_{0}\geq 0$ and *φ*, *ψ*, *η*.

It was demonstrated in [13, 23], that the zero initial functions

2.5 $$ x(\xi )=0,\qquad x^{\prime }(\xi )=0\quad \mbox{and}\quad x^{\prime \prime }(\xi )=0\quad \mbox{for }\xi < 0, $$

can be considered in stability studies instead of the initial functions (2.3). We consider the following homogeneous equation:

2.6 $$ x^{\prime \prime \prime } ( t ) +\sum_{i=0}^{2}\sum _{j=1}^{m}p_{ij}(t)x^{(i)} \bigl(t-\tau _{ij}(t)\bigr)=0,\quad t\in [0,\infty ), $$

and the corresponding non-homogeneous equation

2.7 $$ x^{\prime \prime \prime } ( t ) +\sum_{i=0}^{2}\sum _{j=1}^{m}p_{ij}(t)x^{(i)} \bigl(t-\tau _{ij}(t)\bigr)=f(t),\quad t\in [0,\infty ), $$

with the initial functions (2.5). Equation (2.6) is homogeneous in the sense of the theory of ordinary differential equations: its fundamental system is three-dimensional and the known representations of solutions hold (see [23] or [1], p. 477).

### Definition 2.2

The function $c(t,s)$ satisfying as a function of *t* for every fixed $s\in [0,\infty )$ the equation

2.8 $$ x^{\prime \prime \prime } ( t ) +\sum_{i=0}^{2}\sum _{j=1}^{m}p_{ij}(t)x^{(i)} \bigl(t-\tau _{ij}(t)\bigr)=0,\quad t\in [s,\infty ), $$

where

2.9 $$ x^{(i)}(\xi )=0\quad \mbox{for }\xi < s, i=0,1,2, $$

and the initial conditions $c(s,s)=0$, $c_{t}^{\prime}(s,s)=0$, $c_{tt}^{\prime \prime }(s,s)=1$, is called the Cauchy function of Eq. (2.7).

The solution of non-homogeneous equation (2.7) with the initial conditions $x(0)=0$, $x^{\prime }(0)=0$, $x^{\prime \prime }(0)=0$ can be presented in the form (see [23] or [1], p. 478)

2.10 $$ x(t)= \int _{0}^{t}c(t,s)f(s)\,ds. $$

Note the classical Bohl–Perron theorem [1, 23].

### Lemma 2.1

*If a solution* $x(t)$ *of Eq*. (2.7) *with the initial functions* (2.5) *and its derivatives* $x^{\prime }(t)$ *and* $x^{\prime \prime }(t)$ *are bounded for every essentially bounded function* $f(t)$ *for* $t\in [0,\infty )$, *then Eq*. (2.1) *is exponentially stable*.

## Cauchy function of an autonomous third-order ordinary differential equation

In this section we construct the Cauchy function of the equation

3.1 $$ x^{\prime \prime \prime }(t)+Ax^{\prime \prime }(t)+Bx^{\prime }(t)+Cx(t)=0, $$

where *A*, *B* and *C* are constants according to Definition 2.2. Its characteristic equation is

3.2 $$ k^{3}+Ak^{2}+Bk+C=0, $$

and all real parts of all its roots are negative according to the classical Hurwitz theorem if and only if

3.3 $$ A>0,\qquad B>0,\qquad C>0\quad \mbox{and}\quad AB>C. $$

It will be assumed below that this condition is fulfilled. All possible cases for the roots are the following:

3.4 $$ \begin{aligned} &\mbox{(1) } k_{1},k_{2},k_{3} \mbox{ are real and different }k_{1}\neq k_{2},k_{2} \neq k_{3},k_{3}\neq k_{1}\mbox{,} \\ &\mbox{(2) } k_{1},k_{2},k_{3}\mbox{ are real with a pair of equal ones }k_{1}=k_{2}\neq k_{3}, \\ &\mbox{(3) } \mbox{three multiple real roots }k_{1}=k_{2}=k_{3}, \\ &\mbox{(4) } \mbox{a pair of complex roots }k_{1,2}=\alpha \pm \beta i\text{ and a real root }k_{3}. \end{aligned} $$

In every of these cases, the Cauchy function $W(t,s)$ of Eq. (3.1) could be constructed according to Definition 2.2. Actually, we can solve the third-order autonomous ordinary differential Eq. (3.1) with the initial conditions $x(s)=0$, $x^{\prime }(s)=0$, $x^{\prime \prime }(s)=1$. Taking this for every one of the cases (1)–(4), we obtain Lemmas 3.1–3.4 below.

The Hurwitz theorem guarantees the exponential stability of system (3.1), and, according to Definition 2.2 explaining the construction of the Cauchy function, the exponential estimates of the Cauchy function $W(t,s)$ and its derivatives $W_{t}^{\prime }(t,s),W_{tt}^{\prime \prime }(t,s),W^{\prime \prime \prime }_{\textit{ttt}}(t,s)$ [13, 23]. It is clear that in this case there exist the finite values

3.5 $$\begin{aligned}& \begin{aligned} &w_{0}=\sup_{t\geq 0} \int _{0}^{t} \bigl\vert W(t,s) \bigr\vert \,ds,\qquad w_{1}=\sup_{t\geq 0} \int _{0}^{t} \bigl\vert W_{t}^{\prime }(t,s) \bigr\vert \,ds, \\ &w_{2}=\sup_{t\geq 0} \int _{0}^{t} \bigl\vert W_{tt}^{\prime \prime }(t,s) \bigr\vert \,ds,\qquad w_{3}=\sup_{t\geq 0} \int _{0}^{t} \bigl\vert W_{\textit{ttt}}^{\prime \prime \prime }(t,s) \bigr\vert \,ds. \end{aligned} \end{aligned}$$

Let us start with the case (1) of three different real roots.

### Lemma 3.1

*Let condition* (3.3) *be fulfilled*, *then*, *in the case of* (1) *in* (3.4), *the Cauchy function of Eq*. (3.1) *is of the form*

3.6 $$ W(t,s)=c_{1}e^{k_{1}(t-s)}+c_{2}e^{k_{2}(t-s)}+c_{3}e^{k_{3}(t-s)}, $$

*where*

3.7 $$ \operatorname{col} \{ c_{1},c_{2},c_{3} \} =Q^{-1} \operatorname{col} \{ 0,0,1 \} \quad \textit{and}\quad Q=\left ( \textstyle\begin{array}{@{}c@{\quad}c@{\quad}c@{}} 1 & 1 & 1 \\ k_{1} & k_{2} & k_{3} \\ k_{1}^{2} & k_{2}^{2} & k_{3}^{2}\end{array}\displaystyle \right ) . $$

### Example 3.1

Consider the equation

3.8 $$ x^{\prime \prime \prime }(t)+6x^{\prime \prime }(t)+11x^{\prime }(t)+6x(t)=0. $$

Solving the characteristic equation

3.9 $$ k^{3}+6k^{2}+11k+6=0, $$

we obtain $k_{1}=-1$, $k_{2}=-2$, $k_{3}=-3$. The matrix *Q* is of the form

3.10 $$ Q=\left ( \textstyle\begin{array}{@{}c@{\quad}c@{\quad}c@{}} 1 & 1 & 1 \\ -1 & -2 & -3 \\ 1 & 4 & 9 \end{array}\displaystyle \right ) , $$

and $c_{1}=\frac{1}{2}$, $c_{2}=-1$, $c_{3}=\frac{1}{2}$. The Cauchy function $W(t,s)$ of Eq. (3.1) and its derivatives with respect to the variable *t* are the following:

3.11 $$ \begin{aligned} &W(t,s)=\frac{1}{2}e^{-(t-s)}-e^{-2(t-s)}+ \frac{1}{2}e^{-3(t-s)}, \\ &W_{t}^{\prime }(t,s)=-\frac{1}{2}e^{-(t-s)}-2e^{-2(t-s)}+ \frac{3}{2}e^{-3(t-s)}, \\ &W_{tt}^{\prime \prime }(t,s)=\frac{1}{2}e^{-(t-s)}-4e^{-2(t-s)}+ \frac{9}{2}e^{-3(t-s)}, \\ &W_{\textit{ttt}}^{\prime \prime \prime }(t,s)=-\frac{1}{2}e^{-(t-s)}+8e^{-2(t-s)}- \frac{27}{2}e^{-3(t-s)}, \end{aligned} $$

and after the integration with respect to *s*, we can obtain the inequalities

3.12 $$ w_{0}\leq \frac{7}{6},\qquad w_{1}\leq 2,\qquad w_{2}\leq 4,\qquad w_{3}\leq 9. $$

Consider now the case (2) in (3.4) of two multiple roots.

### Lemma 3.2

*Let condition* (3.3) *be fulfilled*, *then*, *in the case of* (2) *in Eq*. (3.4), *the Cauchy function of Eq*. (3.1) *is of the form*

3.13 $$ W(t,s)=c_{1}e^{k_{1}(t-s)}+c_{2}te^{k_{1}(t-s)}+c_{3}e^{k_{3}(t-s)}, $$

*where*

3.14 $$ \operatorname{col} \{ c_{1},c_{2},c_{3} \} =Q^{-1} \operatorname{col} \{ 0,0,1 \} \quad \textit{and}\quad Q=\left ( \textstyle\begin{array}{@{}c@{\quad}c@{\quad}c@{}} 1 & 0 & 1 \\ k_{1} & 1 & k_{3} \\ k_{1}^{2} & 2k_{1} & k_{3}^{2} \end{array}\displaystyle \right ) . $$

In this case we obtain

3.15 $$ c_{1}=-\frac{1}{(k_{1}-k_{3})^{2}},\qquad c_{2}=\frac{1}{k_{1}-k_{3}}, \qquad c_{3}=\frac{1}{(k_{1}-k_{3})^{2}}, $$

and

3.16 $$\begin{aligned}& w_{0}\leq \frac{1}{ \vert k_{1} \vert (k_{1}-k_{3})^{2}}+\frac{1}{ \vert k_{1}-k_{3} \vert k_{1}^{2}}+\frac{1}{ \vert k_{3} \vert (k_{1}-k_{3})^{2}}, \end{aligned}$$

3.17 $$\begin{aligned}& w_{1}\leq \frac{2}{(k_{1}-k_{3})^{2}}+\frac{2}{ \vert k_{1}-k_{3} \vert \vert k_{1} \vert }, \end{aligned}$$

3.18 $$\begin{aligned}& w_{2}\leq \frac{ \vert k_{1} \vert + \vert k_{3} \vert }{(k_{1}-k_{3})^{2}}+\frac{3}{ \vert k_{1}-k_{3} \vert }, \end{aligned}$$

3.19 $$\begin{aligned}& w_{3}\leq \frac{ \vert k_{1} \vert ^{2}+ \vert k_{3} \vert ^{2}}{(k_{1}-k_{3})^{2}}+\frac{4 \vert k_{1} \vert }{ \vert k_{1}-k_{3} \vert }. \end{aligned}$$

Consider now the case (3) in (3.4) of three multiple roots $k_{1}=k_{2}=k_{3}$.

### Lemma 3.3

*Let condition* (3.3) *be fulfilled*, *then*, *in the case of* (3) *in* (3.4), *the Cauchy function of Eq*. (3.1) *is of the form*

3.20 $$ W(t,s)=\frac{ ( t-s ) ^{2}}{2}e^{k_{1}(t-s)}, $$

*and*

3.21 $$\begin{aligned}& w_{0}\leq \frac{2}{ \vert k_{1} \vert ^{3}},\qquad w_{1}\leq \frac{3}{k_{1}^{2}}, \qquad w_{2}\leq \frac{3}{ \vert k_{1} \vert } + \frac{1+2 \vert k_{1} \vert }{k_{1}^{2}}, \end{aligned}$$

3.22 $$\begin{aligned}& w_{3}\leq \frac{1+3 \vert k_{1} \vert }{ \vert k_{1} \vert }+\frac{2 ( \vert k_{1} \vert +4 \vert k_{1} \vert ^{2} ) }{ \vert k_{1} \vert ^{2}}+2. \end{aligned}$$

Consider now the case (4) in (3.4) of one real root $k_{1}$ and two complex roots $k_{2}=\alpha +i\beta $, $k_{3}=\alpha -i\beta $, where we suppose below that $\beta >0$ without loss of generality.

### Lemma 3.4

*Let condition* (3.3) *be fulfilled*, *then*, *in the case of* (4) *in* (3.4), *the Cauchy function of Eq*. (3.1) *is of the form*

3.23 $$ W(t,s)=c_{1}e^{k_{1}(t-s)}+c_{2}e^{\alpha (t-s)}\cos \beta (t-s)+c_{3}e^{\alpha (t-s)}\sin \beta (t-s), $$

*where*

3.24 $$ \operatorname{col} \{ c_{1},c_{2},c_{3} \} =Q^{-1} \operatorname{col} \{ 0,0,1 \} \quad \textit{and}\quad Q=\left ( \textstyle\begin{array}{@{}c@{\quad}c@{\quad}c@{}} 1 & 1 & 0 \\ k_{1} & \alpha & \beta \\ k_{1}^{2} & \alpha ^{2}-\beta ^{2} & 2\alpha \beta \end{array}\displaystyle \right ) . $$

*In this case*

3.25 $$ c_{1}=\frac{1}{\beta ^{2}+ ( \alpha -k_{1} ) ^{2}},\qquad c_{2}=-\frac{1}{\beta [ \beta ^{2}+ ( \alpha -k_{1} ) ^{2} ] }, \qquad c_{3}=\frac{\alpha -k_{1}}{\beta [ \beta ^{2}+ ( \alpha -k_{1} ) ^{2} ] }, $$

*and*

3.26 $$ w_{0}\leq \frac{1}{\beta ^{2}+ ( \alpha -k_{1} ) ^{2}} \biggl\{ \frac{1}{ \vert k_{1} \vert } + \frac{1}{ \vert \alpha \vert } +\frac{ \vert k_{1}-\alpha \vert }{ \vert \alpha \beta \vert } \biggr\} . $$

## Stability of third-order delay equations

Consider the following equation:

4.1 $$\begin{aligned}& x^{\prime \prime \prime } ( t )+\sum_{i=0}^{2}\sum _{j=1}^{m}p_{ij}(t)x^{(i)} \bigl(t-\tau _{ij}(t)\bigr)=f(t),\quad t\in[0,\infty ), \\& x^{(i)}(\xi )=0\quad \mbox{for }\xi < 0, i=0,1,2. \end{aligned}$$

Denote by $a_{2j}$, $b_{1j}$ and $c_{0j}$ the average values of the coefficients $p_{2j}(t)$, $p_{1j}(t)$, $p_{0j}(t)$, respectively, and $\Delta a_{2j}(t)=p_{2j}(t)-a_{2j}$, $\Delta b_{1j}(t)=p_{1j}(t)-b_{1j}$, $\Delta c_{0j}(t)=p_{0j}(t)-c_{0j}$ for $j=1,\ldots,m$.

Rewrite Eq. (4.1) in the form

4.2 $$\begin{aligned} &x^{\prime \prime \prime } ( t ) +\sum_{j=1}^{m} \bigl(a_{2j}+\Delta a_{2j}(t)\bigr)x^{\prime \prime }\bigl(t-\tau _{2j}(t)\bigr) \\ &\quad {}+\sum_{j=1}^{m} \bigl(b_{1j}+\Delta b_{1j}(t)\bigr)x^{\prime }\bigl(t-\tau _{1j}(t)\bigr) \\ &\quad {}+\sum_{j=1}^{m} \bigl(c_{0j}+\Delta c_{0j}(t)\bigr)x\bigl(t-\tau _{0j}(t)\bigr)=f(t),\quad t\in [0,\infty), \\ &x^{(i)}(\xi )=0\quad \mbox{for }\xi < 0, i=0,1,2. \end{aligned}$$

To connect Eq. (4.2) with (3.1) let us set

4.3 $$ A=\sum_{j=1}^{m}a_{2j},\qquad B= \sum_{j=1}^{m}b_{1j},\qquad C=\sum _{j=1}^{m}c_{0j}. $$

Denote $\tau _{ij}^{\ast }=\operatorname{esssup}_{t\geq 0}\tau _{ij}(t)$, $\Delta a_{2j}^{\ast }=\operatorname{esssup}_{t\geq 0} \vert \Delta a_{2j}(t) \vert $, $\Delta b_{1j}^{\ast }=\operatorname{esssup}_{t\geq 0} \vert \Delta b_{1j}(t) \vert $, $\Delta c_{0j}^{\ast }=\operatorname{esssup}_{t\geq 0} \vert \Delta c_{0j}(t) \vert $, $j=1,\ldots,m$, $i=0,1,2$, and

4.4 $$\begin{aligned} q&=\sum_{j=1}^{m} \vert a_{2j} \vert \tau _{2j}^{\ast } \{ w_{3}+1 \}\,ds+\sum _{j=1}^{m} \vert b_{1j} \vert \tau _{1j}^{\ast }w_{2}+\sum _{j=1}^{m} \vert c_{0j} \vert \tau _{0j}^{\ast }w_{1} \\ &\quad {}+ \sum_{j=1}^{m}\Delta a_{2j}^{\ast }w_{2}+\sum_{j=1}^{m} \Delta b_{1j}^{\ast }w_{1}+\sum _{j=1}^{m}\Delta c_{0j}^{\ast }(t)w_{0}. \end{aligned}$$

It is clear that the choice of the parameters $w_{0}$, $w_{1}$, $w_{2}$ and $w_{3} $ depends on the case (1), (2), (3) and (4) in which the “constant parts” of the coefficients *A*, *B* and *C* of the given Eq. (4.2) are defined by (4.3).

Consider

4.5 $$\begin{aligned} &x^{\prime \prime \prime } ( t ) +\sum_{j=1}^{m} \bigl(a_{2j}+\Delta a_{2j}(t)\bigr)x^{\prime \prime }\bigl(t-\tau _{2j}(t)\bigr) \\ &\quad {}+\sum_{j=1}^{m} \bigl(b_{1j}+\Delta b_{1j}(t)\bigr)x^{\prime }\bigl(t-\tau _{1j}(t)\bigr) \\ &\quad {}+\sum_{j=1}^{m} \bigl(c_{0j}+\Delta c_{0j}(t)\bigr)x\bigl(t-\tau _{0j}(t)\bigr)=0,\quad t\in [0,\infty ), \\ &x^{(i)}(\xi )=0\qquad \mbox{for }\xi < 0, i=0,1,2. \end{aligned}$$

### Theorem 4.1

*If the Hurwitz condition* (3.3) *for* *A*, *B*, *C* *defined by* (4.3) *is fulfilled and* *q*, *defined by Eq*. (4.4), *satisfies the inequality* $q<1$, *then Eq*. (4.5) *is exponentially stable*.

### Remark 4.1

If all the coefficients are constants and $\Delta a_{2j}^{\ast }=\Delta b_{1j}^{\ast }=\Delta c_{0j}^{\ast }=0$, $j=1,\ldots,m$, then

4.6 $$ q=\sum_{j=1}^{m} \vert a_{2j} \vert \tau _{2j}^{\ast } \{ w_{3}+1 \}\,ds+\sum _{j=1}^{m} \vert b_{1j} \vert \tau _{1j}^{\ast }w_{2}+\sum _{j=1}^{m} \vert c_{0j} \vert \tau _{0j}^{\ast }w_{1}. $$

It is clear now that $q<1$ if the delays are small enough.

We obtain the following fact.

### Corollary 4.1

*If the Hurwitz condition* (3.3) *for* *A*, *B*, *C* *defined by* (4.3) *is fulfilled*, *the delays* $\tau _{ij}^{\ast }$ *and* $\Delta a_{2j}^{\ast }$, $\Delta b_{1j}^{\ast }$, $\Delta c_{0j}^{\ast }$ *for* $j=1,\ldots,m$, $i=0,1,2$, *are sufficiently small*, *then Eq*. (4.5) *is exponentially stable*.

### Example 4.1

Consider the equation

4.7 $$\begin{aligned} &x^{\prime \prime \prime }(t)+6x^{\prime \prime }\bigl(t-\tau _{2}(t) \bigr)+11x^{\prime }\bigl(t-\tau _{1}(t)\bigr)+6x\bigl(t-\tau _{0}(t)\bigr)=0, \\ &x^{(i)}(\xi )=0\quad \mbox{for }\xi < 0, i=0,1,2. \end{aligned}$$

In this case Theorem 4.1 and estimates (3.12) in Example 3.1 imply that

4.8 $$ q=60\tau _{2j}^{\ast }+44\tau _{1j}^{\ast }+12 \tau _{0j}^{\ast }. $$

Denoting $X=\tau _{2}$, $Y=\tau _{1}$, $Z=\tau _{0}$, we obtain a simple geometrical interpretation of this result: Eq. (4.7) is exponentially stable if the point $M(\tau _{2}(t),\tau _{1}(t),\tau _{0}(t))$ for every $t\geq 0$ is inside the pyramid formed by the planes $X=0$, $Y=0$, $Z=0$ and $60X+44Y+12Z=1$. The last plane can be constructed as one having the intersections with the axes at the points $( \frac{1}{60},0,0 ) $, $( 0,\frac{1}{44},0 ) $ and $( 0,0,\frac{1}{12} ) $.

### Example 4.2

Consider the equation

4.9 $$\begin{aligned}& x^{\prime \prime \prime }(t)+\bigl(6+\Delta a(t)\bigr)x^{\prime \prime }\bigl(t-\tau _{2}(t)\bigr) \\& \quad {}+\bigl(11+\Delta b(t)\bigr))x^{\prime }\bigl(t-\tau _{1}(t)\bigr)+\bigl(6+\Delta c(t)\bigr)x\bigl(t-\tau _{0}(t)\bigr)=0, \end{aligned}$$

where

4.10 $$ \tau _{2}(t)\leq \frac{1}{240},\qquad \tau _{1}(t)\leq \frac{1}{176},\qquad \tau _{0}(t)\leq \frac{1}{48}. $$

From Theorem 4.1 we obtain the following test of exponential stability:

4.11 $$ \frac{\Delta a^{\ast }}{\frac{1}{16}}+\frac{\Delta b^{\ast }}{\frac{1}{8}}+\frac{\Delta c^{\ast }}{\frac{3}{14}}< 1. $$

Denoting $X=\Delta a^{\ast }$, $Y=\Delta b^{\ast }$, $Z=\Delta c^{\ast }$, we obtain a simple geometrical interpretation of this result: Eq. (4.9) under condition (4.10) is exponentially stable if the point $M(\Delta a(t),\Delta b(t),\Delta c(t))$ for every $t\geq 0$ is inside the pyramid formed by the planes $X=0$, $Y=0$, $Z=0$ and $\frac{X}{\frac{1}{16}}+\frac{Y}{\frac{1}{8}}+\frac{Z}{\frac{3}{14}}=1$. The last plane can be constructed as one having the intersections with the axes at the points $( \frac{1}{16},0,0 ) $, $( 0,\frac{1}{8},0 ) $ and $( 0,0,\frac{3}{14} ) $.

## Proofs

### Proof of Theorem 4.1

Let us assume first that $t-\tau _{ij}(t)\geq 0$ for $i=0,1,2$, $j=1,\ldots,m$, $t\geq 0$. Rewrite Eq. (4.2) in the form

5.1 $$\begin{aligned} &x^{\prime \prime \prime } ( t ) +\sum_{j=1}^{m}a_{2j}x^{\prime \prime }(t)+ \sum_{j=1}^{m}b_{1j}x^{\prime }(t)+ \sum_{j=1}^{m}c_{0j}x(t) \\ &\quad {}+ \sum_{j=1}^{m} \bigl(a_{2j}+\Delta a_{2j}(t)\bigr)x^{\prime \prime }\bigl(t-\tau _{2j}(t)\bigr)-\sum_{j=1}^{m}a_{2j}x^{\prime \prime }(t) \\ &\quad {}+ \sum_{j=1}^{m} \bigl(b_{1j}+\Delta b_{1j}(t)\bigr)x^{\prime }\bigl(t-\tau _{1j}(t)\bigr)-\sum_{j=1}^{m}b_{1j}x^{\prime }(t) \\ &\quad {} + \sum_{j=1}^{m} \bigl(c_{0j}+\Delta c_{0j}(t)\bigr)x\bigl(t-\tau _{0j}(t)\bigr)-\sum_{j=1}^{m}c_{0j}x(t)=f(t), \quad t\in [ 0,\infty ). \end{aligned}$$

We can rewrite Eq. (5.1) in the form

5.2 $$\begin{aligned} &x^{\prime \prime \prime } ( t ) +Ax^{\prime \prime }(t)+Bx^{\prime }(t)+Cx(t) \\ &\quad = \sum_{j=1}^{m}a_{2j} \int _{t-\tau _{2j}(t)}^{t}x^{\prime \prime \prime }(s)\,ds-\sum _{j=1}^{m}\Delta a_{2j}(t)x^{\prime \prime } \bigl(t-\tau _{2j}(t)\bigr) \\ &\quad \quad {}+\sum_{j=1}^{m}b_{1j} \int _{t-\tau _{1j}(t)}^{t}x^{\prime \prime }(s)\,ds-\sum _{j=1}^{m}\Delta b_{1j}(t)x^{\prime } \bigl(t-\tau _{1j}(t)\bigr) \\ &\quad\quad {}+ \sum_{j=1}^{m}c_{0j} \int _{t-\tau _{1j}(t)}^{t}x^{\prime }(s)\,ds+\sum _{j=1}^{m}\Delta c_{0j}(t)x(t-\tau _{0j}(t)+f(t). \end{aligned}$$

Let us use the Azbelev *W*-transform [23],

5.3 $$ x(t)= \int _{0}^{t}W(t,s)z ( s )\,ds, $$

where $z\in L_{\infty }$ ($L_{\infty }$ is the space of essentially bounded functions $z:[0,\infty )\rightarrow\mathbb{R}$), $W(t,s)$ is the Cauchy function of Eq. (3.1). It is clear that Eq. (5.3) is the representation of the solution of the initial value problem

5.4 $$\begin{aligned}& x^{\prime \prime \prime }(t)+Ax^{\prime \prime }(t)+Bx^{\prime }(t)+Cx(t)=z(t), \quad t \in [0,\infty ), \end{aligned}$$

5.5 $$\begin{aligned}& x(0)=0,\qquad x^{\prime }(0)=0,\qquad x^{\prime \prime }(0)=0. \end{aligned}$$

Differentiating (5.3), we can find

5.6 $$\begin{aligned}& x^{\prime }(t)= \int _{0}^{t}W_{t}^{\prime }(t,s)z ( s)\,ds, \qquad x^{\prime \prime }(t)= \int _{0}^{t}W_{tt}^{\prime \prime }(t,s)z ( s )\,ds, \end{aligned}$$

5.7 $$\begin{aligned}& x^{\prime \prime \prime }(t)= \int _{0}^{t}W_{\textit{ttt}}^{\prime \prime \prime }(t,s)z ( s )\,ds+z(t). \end{aligned}$$

After the substitution (5.3), (5.6), (5.7) into (5.2) we obtain the equation

5.8 $$ z ( t ) =Kz ( t ) +f ( t ), $$

where the operator $K:L_{\infty }\rightarrow L_{\infty }$ is defined as follows:

5.9 $$\begin{aligned} Kz(t)&= \sum_{j=1}^{m}a_{2j} \int _{t-\tau _{2j}(t)}^{t} \biggl\{ \int _{0}^{s}W_{sss}^{\prime \prime \prime }(s,\eta )z ( \eta )\,d\eta +z(s) \biggr\} \,ds \\ &\quad {}-\sum_{j=1}^{m}\Delta a_{2j}(t) \int _{0}^{t-\tau _{2j}(t)}W_{tt}^{\prime \prime }\bigl(t- \tau _{2j}(t),s\bigr)z ( s )\,ds \\ &\quad {}+ \sum_{j=1}^{m}b_{1j} \int _{t-\tau _{1j}(t)}^{t} \biggl\{ \int _{0}^{s}W_{ss}^{\prime \prime }(s,\eta )z ( \eta )\,d\eta \biggr\} \,ds \\ &\quad {} -\sum_{j=1}^{m}\Delta b_{1j}(t) \int _{0}^{t-\tau _{1j}(t)}W_{t}^{\prime }\bigl(t- \tau _{1j}(t),s\bigr)z ( s )\,ds \\ &\quad {}+ \sum_{j=1}^{m}c_{0j} \int _{t-\tau _{0j}(t)}^{t} \biggl\{ \int _{0}^{t}W_{s}^{\prime }(s,\eta )z ( \eta )\,d\eta \biggr\} \,ds \\ &\quad {}+ \sum_{j=1}^{m}\Delta c_{0j}(t) \int _{0}^{t-\tau _{0j}(t)}W\bigl(t-\tau _{0j}(t),s \bigr)z ( s )\,ds. \end{aligned}$$

The condition $q<1$, where *q* is defined by Eq. (4.4), implies that the norm $\Vert K \Vert $ of the operator $K:L_{\infty }\rightarrow L_{\infty }$ is less than one and this guarantees the action and boundedness of the operator $(I-K)^{-1}=I-K-K^{2}+K^{3}+\cdots$ from $L_{\infty }$ to $L_{\infty }$. It is clear now that, for every bounded right-hand side *f*, the solution *z* of Eq. (5.8) is bounded. From the Hurwitz condition (3.3) on Eq. (3.1) it follows that the solution $x(t)$ and its derivatives $x^{\prime }(t)$ and $x^{\prime \prime }(t)$ defined by formulas (5.3) and (5.6) are bounded on the semiaxis $t\in [0,\infty ) $ for any bounded right-hand side *f*. The Bohl–Perron theorem formulated in Lemma 2.1 (see also [23], p. 93 or [1], p. 500 in a more general formulation) claims that boundedness of solutions of Eq. (4.2) for all bounded right-hand sides *f* is equivalent to the exponential stability of Eq. (4.5). Thus the reference to the Bohl–Perron theorem completes this part of the proof.

If we do not assume that $t-\tau _{ij}(t)\geq 0$ for $i=0,1,2$, $j=1,\ldots,m$, $t\geq 0$, we can extend the coefficients on the interval $[-\tau ,0)$, where $\tau =\operatorname{esssup}_{t\geq 0}\tau _{ij}(t)$, as follows: $\tau _{ij}(t)\equiv 0, p_{2j}(t)\equiv \sum_{j=1}^{m}a_{2j},p_{1j}(t)\equiv \sum_{j=1}^{m}b_{1j}$ and $p_{1j}(t)\equiv \sum_{j=1}^{m}c_{0j}$ and consider Eq. (4.1) on the interval $[-\tau ,\infty )$. Passing now to Eqs. (4.2) and (4.5) on this interval $[-\tau ,\infty )$, we can repeat the whole proof. This remark completes the proof of Theorem 4.1. □

## Conclusion, discussion and some topics for future research

In this paper we propose a general algorithm for stabilization of third-order differential Eq. (1.1) by the delay feedback control

6.1 $$ u(t)=-\sum_{i=0}^{2}\sum _{j=1}^{m}p_{ij}(t)x^{(i)}\bigl(t- \tau _{ij}(t)\bigr). $$

If the delays $\tau _{ij}(t)$ and “oscillations” of the coefficients $\vert \Delta a_{2j}(t) \vert $, $\vert \Delta b_{1j}(t) \vert $, $\vert \Delta c_{0j}(t) \vert $ are small enough, then Eq. (4.5) becomes exponentially stable and stabilization is achieved. In the model of ship stabilization, which can be described by the third-order delay equation with constant coefficients, a sufficient smallness of the delays looks very natural from the mechanical point of view.

Note that a similar idea for stability studies of the second-order delay differential equations was proposed first in [26], developed then in [27] and the exact estimates of the integrals of the Cauchy functions (i.e. of $w_{0}$, $w_{1}$, $w_{2}$) for second-order equations were obtained in [28].

The following question for future research could be considered: is it possible to obtain the exponential stability in the case of $A=0$ and/or $B=0?$ A question of this sort had a long history for second-order delay equations. Myshkis considered the equation $x^{\prime \prime }(t)+px(t-\tau )=0$ and, analyzing the roots of characteristic equation, proved instability of this equation for every positive constants *p* and *τ* (see [29] Chapter III, Section 16, pp. 105–106). In [30] it was proven that all solutions of the equation $x^{\prime \prime }(t)+px(t-\tau (t))=0$ with every positive constant *p* and nonnegative $\tau (t)$ are bounded if and only if $\int ^{\infty }\tau (t)\,dt<\infty $. It was considered impossible to obtain exponential stability of second-order delay equations without damping terms for the delay satisfying the inequality $\tau (t)>\varepsilon $ for every positive *ε*. Using an analysis of the roots of the characteristic equations, first results on the stability of the equation $x^{\prime \prime }(t)+ax(t)-bx(t-\tau )=0$ (*a*, *b* and *τ* are constant parameters) were obtained in [2, 4, 21]. In the case of variable coefficients and delays, results on the exponential stability of second-order delay equation

6.2 $$ x^{\prime \prime } ( t ) +\sum_{j=1}^{m}p_{j}(t)x \bigl(t-\tau _{j}(t)\bigr)=0,\quad t\in [0,\infty ), $$

were obtained first in [3]. An approach to the analysis of the stability of this second-order equation based on estimates of the integrals of the Cauchy functions $w_{0}$, $w_{1}$, $w_{2}$ was proposed in [31]. We suppose that this approach can be developed also in the stability analysis of the third-order delay equation

6.3 $$ x^{\prime \prime \prime } ( t ) +\sum_{j=1}^{m}p_{j}(t)x \bigl(t-\tau _{j}(t)\bigr)=0,\quad t\in [0,\infty ). $$

It is interesting to develop the method proposed in our paper for stability studies of systems of delay equations. Another possible development is to apply our “linear” results to the stability of nonlinear delay differential equations and to obtain, for example, analogous results to the ones obtained in [11, 17, 19].
